# Supplementary material for: The Risk of Hepatitis B Virus Reactivation in Rheumatoid Arthritis Patients Receiving Tocilizumab: A Systematic Review and Meta-Analysis
Source: Viruses. 2024 Jan 3;16(1):78. doi: 10.3390/v16010078 (PMC10820547; doi:10.3390/v16010078)

The Risk of Hepatitis B Virus Reactivation in Rheumatoid Arthritis Patients Receiving Tocilizumab: A Systematic Review and Meta-Analysis_ Supporting information

Ping-Hung Ko, [1,2] Meng Hsuan Kuo[3]*, I-Ting Kao,[3] Chen-Yi Wu[3], Chih-Wei Tseng[1,2]*, and Shih-Chieh Shao[4]

Co-corresponding author:

Chih-Wei Tseng, MD. Email: cwtseng2@gmail.com; dm650504@tzuchi.com.tw
Meng Hsuan Kuo, MS. Email: danms0521@gmail.com;df441865@tzuchi.com.tw

Table S1. PRISMA 2020 checklist

Table S2. Search strategy

Table S3. Quality assessment of the included cohort studies using Newcastle-Ottawa Scale

Table S4. Subgroup analysis of the polled rate of HBVr in HBsAg−/ anti-HBc + rheumatoid arthritis patients without NA prophylaxis treating with tocilizumab

Figure S1. Publication bias analysis by using funnel plot for the risk of HBV reactivation in HBsAg−/ anti-HBc + patients

Figure S2. Sensitivity analysis for the risk of HBV reactivation in HBsAg−/ anti-HBc + patients

Table S1. PRISMA 2020 checklist

Section and Topic	Item #	Checklist item	Location where item is reported
**TITLE**			
Title	1	Identify the report as a systematic review.	P1
**ABSTRACT**			
Abstract	2	See the PRISMA 2020 for Abstracts checklist.	P2
**INTRODUCTION**			
Rationale	3	Describe the rationale for the review in the context of existing knowledge.	P4
Objectives	4	Provide an explicit statement of the objective(s) or question(s) the review addresses.	P5
**METHODS**			
Eligibility criteria	5	Specify the inclusion and exclusion criteria for the review and how studies were grouped for the syntheses.	P6
Information sources	6	Specify all databases, registers, websites, organisations, reference lists and other sources searched or consulted to identify studies. Specify the date when each source was last searched or consulted.	P6
Search strategy	7	Present the full search strategies for all databases, registers and websites, including any filters and limits used.	P6<br>Table S2
Selection process	8	Specify the methods used to decide whether a study met the inclusion criteria of the review, including how many reviewers screened each record and each report retrieved, whether they worked independently, and if applicable, details of automation tools used in the process.	P6
Data collection process	9	Specify the methods used to collect data from reports, including how many reviewers collected data from each report, whether they worked independently, any processes for obtaining or confirming data from study investigators, and if applicable, details of automation tools used in the process.	P6
Data items	10a	List and define all outcomes for which data were sought. Specify whether all results that were compatible with each outcome domain in each study were sought (e.g. for all measures, time points, analyses), and if not, the methods used to decide which results to collect.	P6

Section and Topic	Item #	Checklist item	Location where item is reported
	10b	List and define all other variables for which data were sought (e.g. participant and intervention characteristics, funding sources). Describe any assumptions made about any missing or unclear information.	P6
Study risk of bias assessment	11	Specify the methods used to assess risk of bias in the included studies, including details of the tool(s) used, how many reviewers assessed each study and whether they worked independently, and if applicable, details of automation tools used in the process.	P7
Effect measures	12	Specify for each outcome the effect measure(s) (e.g. risk ratio, mean difference) used in the synthesis or presentation of results.	P7
Synthesis methods	13a	Describe the processes used to decide which studies were eligible for each synthesis (e.g. tabulating the study intervention characteristics and comparing against the planned groups for each synthesis (item #5)).	P7
	13b	Describe any methods required to prepare the data for presentation or synthesis, such as handling of missing summary statistics, or data conversions.	Not necessary
	13c	Describe any methods used to tabulate or visually display results of individual studies and syntheses.	Not necessary
	13d	Describe any methods used to synthesize results and provide a rationale for the choice(s). If meta-analysis was performed, describe the model(s), method(s) to identify the presence and extent of statistical heterogeneity, and software package(s) used.	P7
	13e	Describe any methods used to explore possible causes of heterogeneity among study results (e.g. subgroup analysis, meta-regression).	P7
	13f	Describe any sensitivity analyses conducted to assess robustness of the synthesized results.	P7
Reporting bias assessment	14	Describe any methods used to assess risk of bias due to missing results in a synthesis (arising from reporting biases).	P7
Certainty assessment	15	Describe any methods used to assess certainty (or confidence) in the body of evidence for an outcome.	P7
**RESULTS**			
Study selection	16a	Describe the results of the search and selection process, from the number of records identified in the search to the number of studies included in the review, ideally using a flow diagram.	Figure 1

Section and Topic	Item #	Checklist item	Location where item is reported
			P8
	16b	Cite studies that might appear to meet the inclusion criteria, but which were excluded, and explain why they were excluded.	Table S3
Study characteristics	17	Cite each included study and present its characteristics.	Table 1
Risk of bias in studies	18	Present assessments of risk of bias for each included study.	Table S4
Results of individual studies	19	For all outcomes, present, for each study: (a) summary statistics for each group (where appropriate) and (b) an effect estimate and its precision (e.g. confidence/credible interval), ideally using structured tables or plots.	Figure 2
Results of syntheses	20a	For each synthesis, briefly summarise the characteristics and risk of bias among contributing studies.	P8
	20b	Present results of all statistical syntheses conducted. If meta-analysis was done, present for each the summary estimate and its precision (e.g. confidence/credible interval) and measures of statistical heterogeneity. If comparing groups, describe the direction of the effect.	Table S5
	20c	Present results of all investigations of possible causes of heterogeneity among study results.	P8-9
	20d	Present results of all sensitivity analyses conducted to assess the robustness of the synthesized results.	Figure S2<br>P9
Reporting biases	21	Present assessments of risk of bias due to missing results (arising from reporting biases) for each synthesis assessed.	Figure S1<br>P9-10
Certainty of evidence	22	Present assessments of certainty (or confidence) in the body of evidence for each outcome assessed.	Nil
**DISCUSSION**			
Discussion	23a	Provide a general interpretation of the results in the context of other evidence.	P11-12
	23b	Discuss any limitations of the evidence included in the review.	P13

Section and Topic	Item #	Checklist item	Location where item is reported
	23c	Discuss any limitations of the review processes used.	P13
	23d	Discuss implications of the results for practice, policy, and future research.	P13
**OTHER INFORMATION**			
Registration and protocol	24a	Provide registration information for the review, including register name and registration number, or state that the review was not registered.	INPLASY 202360029
	24b	Indicate where the review protocol can be accessed, or state that a protocol was not prepared.	P6
	24c	Describe and explain any amendments to information provided at registration or in the protocol.	Nil
Support	25	Describe sources of financial or non-financial support for the review, and the role of the funders or sponsors in the review.	P15
Competing interests	26	Declare any competing interests of review authors.	P15
Availability of data, code and other materials	27	Report which of the following are publicly available and where they can be found: template data collection forms; data extracted from included studies; data used for all analyses; analytic code; any other materials used in the review.	Nil

Table S2. Search strategy

Database	Keyword	Date	Results
PubMed	Search: (hepatitis B reactivation OR virus activation OR virus reactivation OR recurrence OR recurrent infection OR reactivation OR HBV OR hepatitis B OR hepatitis B virus OR hepatitis B surface antigen OR hepatitis B antibody OR HBsAg OR anti-HBc OR anti-HBs) AND (anti-interleukin-6 receptor monoclonal antibody OR interleukin-6-receptor inhibitor OR Interleukin 6 inhibitors OR tocilizumab)<br>Filters: Clinical Study, Clinical Trial, Observational Study, Randomized Controlled Trial, Humans	2023.10.13	71
Embase	(hepatitis B reactivation OR virus activation OR virus reactivation OR recurrence OR recurrent infection OR reactivation OR HBV OR hepatitis B OR hepatitis B virus OR hepatitis B surface antigen OR hepatitis B antibody OR HBsAg OR anti-HBc OR anti-HBs) AND (anti-interleukin-6 receptor monoclonal antibody OR interleukin-6-receptor inhibitor OR Interleukin 6 inhibitors OR tocilizumab)	2023.10.13	163
Cochrane Central Register of Controlled Trials	((((((((hepatitis AND b AND reactivation OR virus) AND activation OR virus) AND reactivation OR recurrence OR recurrent) AND infection OR reactivation OR hbv OR hepatitis) AND b OR hepatitis) AND b AND virus OR hepatitis) AND b AND surface AND antigen OR hepatitis) AND b AND antibody OR hbsag OR 'anti hbc' OR 'anti hbs') AND ((('anti interleukin 6' AND receptor AND monoclonal AND antibody OR 'interleukin 6 receptor') AND inhibitor OR interleukin) AND 6 AND inhibitors OR tocilizumab) AND [article]/lim AND [humans]/lim AND [clinical study]/lim	2023.10.13	157

Table S3. Quality assessment of the included cohort studies using the Newcastle-Ottawa Scale[a]

Type of HBV status	First author	Selection				Comparability		Outcome			Total points	Overall RoB[b]
		Q1	Q2	Q3	Q4	Q1	Q2	Q1	Q2	Q3		
HBsAg+	Chen LF 2017	0	1	1	1	0	0	1	0	1	5	High
	Lin 2019	0	1	0	1	0	0	1	1	1	5	High
	Kuo 2021	0	1	1	1	0	0	1	1	1	6	High
HBsAg−/ Anti-HBc+	Mori 2011	0	1	1	1	0	0	1	0	0	4	High
	Nakamura 2016	0	1	1	1	0	0	1	0	0	4	High
	Chen LF 2017	0	1	1	1	0	0	1	0	1	5	High
	Ahn 2018	0	1	1	1	0	0	1	1	1	6	High
	Papalopoulos 2018	0	1	1	1	0	0	1	0	0	4	High
	Tien 2018	0	1	1	1	0	0	1	1	0	5	High
	Carlino 2019	1	1	0	1	1	1	1	1	0	7	Moderate
	Watanabe 2019	0	1	1	1	1	1	1	0	1	7	Moderate
	Chen MH 2021	0	1	1	1	1	1	1	1	0	7	Moderate
	Kuo 2021	0	1	1	1	0	0	1	1	1	6	High

RoB, risk of bias.

[a]The judgments of each item in the selection, comparability, and exposure are as follows:

Selection:

Q1. Representativeness of exposed cohort: 1, truly or somewhat representative of a population-based or multi-center study; 0, selected group of users or lack of description of the derivation of the cohort.

Q2. Selection of non-exposed cohort: 1, drawn from the same community as the exposed cohort; 0, drawn from a different source or lack of description of the derivation of the non-exposed cohort.

Q3. Ascertainment of exposure: 1, secure record or structured interview; 0, written self-report or lack of description of validation.

Q4. Demonstration that outcome of interest was not present at start of study: 1, yes; 0, no.

Comparability:

Q1. Study adjusted for age and sex: 1, yes; 0, no

Q2. study controls for any additional factor: 1, yes; 0, no.

Outcome:

Q1. Assessment of outcome: 1, independent blind assessment, confirmed by medical records or record linkage; 0, self-reported or no description.

Q2. Was follow-up long enough for outcomes to occur: 1, duration of follow-up at least 6 months; 0, duration of follow-up < 6 months.

Q3. Loss to follow-up rate: 1, complete follow-up of loss to follow-up rate less than 20%; 0, loss to follow-up rate more than 20% or no statement.

[b]We considered studies with a score of 9 stars to be at low RoB, studies that scored 7 or 8 stars at moderate RoB, and those that scored 6 stars or less at high RoB.

Table S4. Subgroup analysis of the polled rate of HBVr in HBsAg−/ anti-HBc + rheumatoid arthritis patients without NA prophylaxis treated with tocilizumab

Subgroup	Records (N)	Patients (N)	Polled rate (%)	95% confidence interval (%)	I^2 (%)
**Overall**	10	322	3.3	1.6–6.7	0
**Anti-HBs negative**	4	30	10.9	3.5–29.2	0
**Definition of HBV reactivation**					
HBV DNA reappearance or elevation	5	93	5.9	2.4–14.1	0
HBsAg seroreversion	1	81	0.6	0.0–9.0	0
Both criteria	3	121	1.7	0.4–6.6	0
**Region**					
Asian	8	265	3.7	1.7–7.8	0
Non-Asian	2	57	1.7	0.2–11.1	0
**Risk of bias**					
Moderate	3	133	2.0	0.5–7.8	0
High	7	189	4.0	1.7–9.0	0
**Study design**					
Prospective	2	57	1.9	0.3–12.1	0
Retrospective	8	275	3.7	1.7–7.7	0

Figure S1. Funnel plot analysis of publication bias for the risk of HBV reactivation in HBsAg−/ anti-HBc + patients

(p = 0.04; Egger's test)

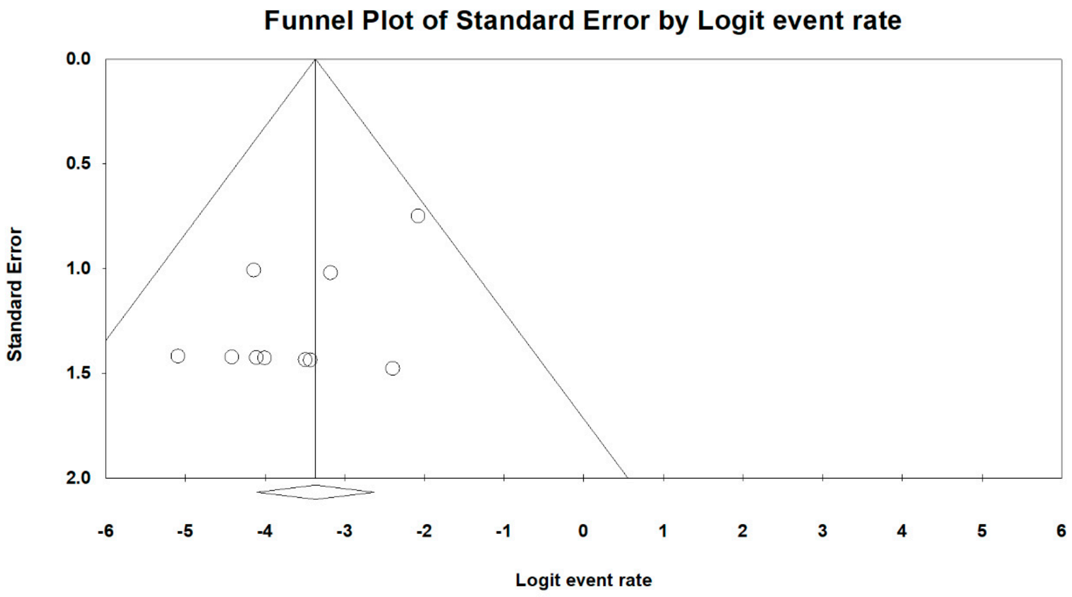

Figure S2. Sensitivity analysis for the risk of HBV reactivation in HBsAg−/ anti-HBc + patients

Study name	Point	Lower limit	Upper limit
Mori 2011	0.031	0.015	0.064
Nakamura 2016	0.022	0.010	0.050
Chen LF 2017	0.036	0.017	0.074
Ahn 2018	0.033	0.016	0.069
Papalopoulos 2018	0.035	0.017	0.072
Tien 2018	0.034	0.016	0.069
Carlino 2019	0.035	0.017	0.072
Watanabe 2019	0.032	0.015	0.069
Chen MH 2021	0.038	0.018	0.078
Kuo 2021	0.038	0.017	0.079
Pooled	0.033	0.016	0.067

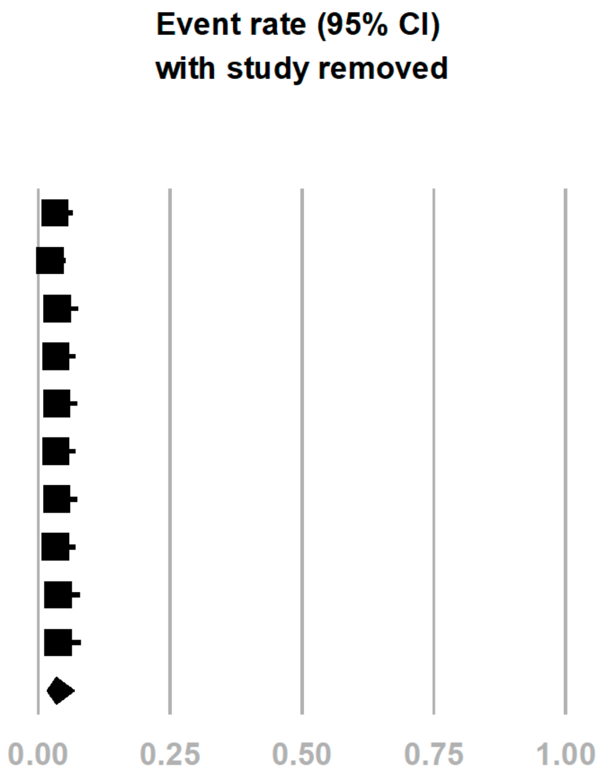

Supplement: Supplementary file 1 [file viruses-16-00078-s001.zip › viruses-2773309-supplementary.pdf]
